# Hypomyelinating disorders in China: The clinical and genetic heterogeneity in 119 patients

**DOI:** 10.1371/journal.pone.0188869

**Published:** 2018-02-16

**Authors:** Haoran Ji, Dongxiao Li, Ye Wu, Quanli Zhang, Qiang Gu, Han Xie, Taoyun Ji, Huifang Wang, Lu Zhao, Haijuan Zhao, Yanling Yang, Hongchun Feng, Hui Xiong, Jinhua Ji, Zhixian Yang, Liping Kou, Ming Li, Xinhua Bao, Xingzhi Chang, Yuehua Zhang, Li Li, Huijuan Li, Zhengping Niu, Xiru Wu, Jiangxi Xiao, Yuwu Jiang, Jingmin Wang

**Affiliations:** 1 Department of Pediatrics, Peking University First Hospital, Beijing, China; 2 Department of Radiology, Peking University First Hospital, Beijing, China; 3 Department of Neurology, First Hospital of Shanxi Medical University, Taiyuan, China; Duke University, UNITED STATES

## Abstract

**Objective:**

Hypomyelinating disorders are a group of clinically and genetically heterogeneous diseases characterized by neurological deterioration with hypomyelination visible on brain MRI scans. This study was aimed to clarify the clinical and genetic features of HMDs in Chinese population.

**Methods:**

119 patients with hypomyelinating disorders in Chinese population were enrolled and evaluated based on their history, clinical manifestation, laboratory examinations, series of brain MRI with follow-up, genetic etiological tests including chromosomal analysis, multiplex ligation probe amplification, Sanger sequencing, targeted enrichment-based next-generation sequencing and whole exome sequencing.

**Results:**

Clinical and genetic features of hypomyelinating disorders were revealed. Nine different hypomyelinating disorders were identified in 119 patients: Pelizaeus-Merzbacher disease (94, 79%), Pelizaeus-Merzbacher-like disease (10, 8%), hypomyelination with atrophy of the basal ganglia and cerebellum (3, 3%), GM1 gangliosidosis (5, 4%), GM2 gangliosidosis (3, 3%), trichothiodystrophy (1, 1%), Pol III-related leukodystrophy (1, 1%), hypomyelinating leukodystrophy type 9 (1, 1%), and chromosome 18q deletion syndrome (1, 1%). Of the sample, 94% (112/119) of the patients were genetically diagnosed, including 111 with mutations distributing across 9 genes including *PLP1*, *GJC2*, *TUBB4A*, *GLB1*, *HEXA*, *HEXB*, *ERCC2*, *POLR3A*, and *RARS* and 1 with mosaic chromosomal change of 46, XX,del(18)(q21.3)/46,XX,r(18)(p11.32q21.3)/45,XX,-18. Eighteen novel mutations were discovered. Mutations in *POLR3A* and *RARS* were first identified in Chinese patients with Pol III-related leukodystrophy and hypomyelinating leukodystrophy, respectively.

**Significance:**

This is the first report on clinical and genetic features of hypomyelinating disorders with a large sample of patients in Chinese population, identifying 18 novel mutations especially mutations in *POLR3A* and *RARS* in Chinese patients, expanding clinical and genetic spectrums of hypomyelinating disorders.

## Introduction

Hypomyelinating disorders (HMDs) are a spectrum of genetic disorders with deficiency in the process of myelination, characterized by a persistent high intensity T2 signal coupled with a mildly hypo-, iso-, or high intensity T1 signal in white matter of an MRI scan[1]. A prevalence rate of 0.78/100,000 and an incidence rate of 1.40/100,000 live births were reported in Japan[2]. Nystagmus and developmental delay often occur in the neonatal or infantile period, and hypotonia, spasticity, extrapyramidal signs, ataxia, or seizures may also be present. Both X-linked and autosomal recessive/dominant gene mutations are identified responsible. HMDs can also result from chromosomal aberrations, such as chromosomal 18q terminal deletion[2–4].

The first HMD to be identified was Pelizaeus-Merzbacher disease (PMD), in 1885, and more than twenty HMDs has been discovered in the past 20 years (Table 1). The hypomyelination pattern was first defined in 2009, and MRI and next generation sequencing were reported important tools for identifying HMDs [3, 5, 6]. Up to date, seldom general analysis on HMDs was reported except one in 2014 [2], and more reports were needed to illuminate the HLDs features, especially in Chinese population without analysis yet. In this study, we examine 119 Chinese patients with HMDs to elucidate the clinical and genetic features of HMDs in Chinese population, and to facilitate correct genetic consulting and prenatal diagnosis for the families affected.

**Table 1:** Characteristics of hypomyelinating disorders.

Disorders	OMIM	Genes	Inheritance pattern	Clinical features	MRI features	Reference
HLD1/Pelizaeus-Merzbacher Disease (PMD)	312080	*PLP1*	XR	Nystagmus, developmental delay, hypotonia, ataxia, extrapyramidal signs, spastic paraplegia	Homogeneous hypomyelination in white matter; atrophy of corpus callosum; cerebellar atrophy	[7]
HLD2/Pelizaeus-Merzbacher-like Disease (PMLD)	608804	*GJC2*	AR	Similar to classic phenotype of PMD	Similar to PMD	[7]
HLD3	260600	*AIMP1*	AR	Severe neurological deterioration, severe developmental delay, microcephaly, dysmorphia, spastic paraplegia, nystagmus	Hypomyelination; brain atrophy especially corpus callosum	[8, 9]
HLD4/Mitochondrial hsp60 chaperonopathy	612233	*HSPD1*	AR	Developmental delay, spasticity, hypotonia, nystagmus, seizures	Homogeneous hypomyelination, atrophy in corpus callosum, cerebrum, brainstem, cerebellum	[10, 11]
HLD5/Hypomyelination and congenital cataract (HCC)	610532	*FAM126A*	AR	Cataract, development delay, pyramidal and cerebellar dysfunction, muscle weakness and wasting	Hypomyelination with preserved cortex and deep gray matter	[12]
HLD6/Hypomyelination with atrophy of the basal ganglia and cerebellum (HABC)	612438	*TUBB4A*	AD	Developmental delay, hypotonia, nystagmus, extrapyramidal signs, ataxia	Hypomyelination, atrophy of putamen and caudate nucleus, cerebellar and/or cerebral atrophy	[13]
HLD7/HLD8/Pol III-Related Leukodystrophies	607694/614381/616494	*POLR3A/POLR3B/POLR1C*	AR	Developmental and growth abnormality, cerebellum signs, extrapyramidal signs, delayed dentition/hypodontia, delayed puberty	Hypomyelination; cerebellar atrophy; T2 hypointensity in optic radiation, posterior limb of the internal capsule, ventrolateral thalamus, and dentate nucleus	[14, 15]
HLD9	616140	*RARS*	AR	Developmental delay, spasticity, nystagmus, ataxia, microcephaly, pyramidal signs, and extrapyramidal signs	Diffuse hypomyelination in white matter and atrophy of corpus callosum, cerebrum, and cerebellum	[16]
HLD10	616420	*PYCR2*	AR	Developmental delay/regression, microcephaly, spastic paraplegia, seizures, dysmorphic features	Hypomyelination, cerebral atrophy, thin corpus callosum	[17]
HLD12	616683	*VPS11*	AR	Severe motor impairment, visual and hearing loss, intellectual disability, seizures, microcephaly	Hypomyelination; atrophy of corpus callosum	[18, 19]
HLD13	616881	*C11orf73*	AR	Early feeding difficulties, global developmental delay, nystagmus, postnatal progressive microcephaly, truncal hypotonia, lower limb spasticity	Hypomyelination, periventricular white matter changes, periventricular cystic changes	[20]
Hypomyelination with brainstem and spinal cord involvement and leg spasticity (HBSL)	615281	*DARS*	AR	Severe spasticity paraplegia, gobal developmental delay, nystagmus	Hypomyelination, white matter lesions in the cerebrum, brainstem, cerebellum, and spinal cord	[21]
Neurodegeneration due to cerebral folate transport deficiency	613068	*FOLR1*	AR	Folate responsive epilepsy, mental retardation, ataxia	Hypomyelination, cerebellar and parieto-temporal atrophy, calcifications in the lentiform nuclei and peripheral white matter	[22, 23]
PCWH syndrome	609136	*SOX10*	AD	Peripheral neuropathy, central dysmyelination, Waardenburg syndrome, and Hirschsprung disease	Hypomyelination with/without atrophy in cerebrum, cerebellum, brainstem; labyrinthine dysplasia and cochlear nerve aplasia	[24, 25]
Trichothiodystrophy	601675/616390/616395/234050/ 616943/300953	*ERCC2/ERCC3/GTF2H5/MPLKIP/GTF2E2/RNF113A*	AR/XLD	Developmental delay, ichthyosis, photosensitivity, brittle and sparse hair, immunodeficiency	Hypomyelination, central osteosclerosis	[26–31]
Chromosome 18q deletion syndrome	601808	*Chromosome 18q deletion*		Growth and developmental abnormality, dysmorphic features, abnormal neurologic signs, immunological, infectious, endocrinological disorders	Mild hypomyelination	[32]
Cockayne syndrome	133540/216400	*CSB (ERCC6) / CSA (ERCC8)*	AR	Developmental delay, microcephaly, facial malformation, photosensitivity, pigmentary retinopathy, cataracts, sensorineural deafness	Hypomyelination; atrophy of cerebellum and brain stem; calcification in subcortical white matter or putamen	[33]
Fucosidosis	230000	*FUCA1*	AR	Developmental delay, angiokeratoma, neurologic signs, coarse facial features, and dysostosis multiplex	Hypomyelination, high T1 and low T2 signal in globus pallidus, thalamus, and substantia nigra, cerebral and cerebellar atrophy may be prominent in older patients	[34]
GM1 gangliosidosis	230500/230600/230650	*GLB1*	AR	Developmental delay/regression, hepatosplenomegaly, macular cherry-red spots, coarse facies, hypotonia, Mongolian spots, dysostosis multiplex and vertebral changes	Hypomyelination; cerebral atrophy; T2 hyperintensity in basal ganglia(putamen); T2 hypointensity in globi pallidi	[35–37]
GM2 gangliosidosis	272800	*HEXA/HEXB*	AR	Developmental delay/regression, hyperacusis, hypotonia, spasticity, seizures, visual impairment	Diffuse hypomyelination; T2 hypointensity and T1 hyperintensity in thalami	[38]

## Subjects and methods

### Patients

All 119 patients from 117 families were recruited using the criteria of development delay and hypomyelination in brain MRI[2]. Diagnosis was based on hyperintensity in brain white matter in T2WI MRI scans taken in patients older than one year of age, or in those younger than one year of age with a second, follow-up scan six months later[6]. Patients with demyelinating disorders, toxic injuries, infectious and post-infectious white matter damage were excluded[5]. P1-P7, P67-P74, and P96-P102 were from our previous report [39–42]. Informed written consent was obtained from the parents of each patient, and this study was approved by the local ethics committees at Peking University First Hospital.

#### Clinical analysis

All 119 patients (P1–P119), including 100 males and 19 females, were enrolled at the Department of Pediatrics, Peking University First Hospital, between September 2005 and November 2014. Two sibling pairs were included (P97 and P98, P113 and P114). Their ages at the first visit to our clinic ranged from 2 months to 27 years, with a median age of 17 months. Forty-three patients had a positive family history, and consanguinity was not found with all patients’ parents. Clinical materials were collected from all enrolled subjects, including history, clinical manifestation, auditory brainstem response, assessment of lysosome enzymes, and brain MRI scans. MRIs taken in patients older than one year of age were assessed for myelination using T1 and T2 signals on the MRI, according to methods recommended by Steenweg et al.[6].

### Genetic analysis

Genomic DNA was extracted from peripheral venous blood leukocytes from both patients and their families[43], and genetic analysis was performed for all. The genetic analysis methods were chosen based on clinical diagnosis. *HEXA* or *HEXB* sequencing was taken for patients with deficiency of β-galactosidase or hexosaminidase A or B. For patients with PMD, the copy number variation of *PLP1* were detected by multiplex ligation probe amplification (MLPA) using a SALSA MLPA P022 or P071 kit (MRC-Holland, Amsterdam, NH, NL), following the manufacturer’s protocol. The pattern of X-chromosome inactivation (XCI) was evaluated in the female patients with *PLP1* mutations and ratios higher than 80:20 were considered skewed [44][45]. *PLP1* sequencing was used for patients with negative MLPA results and if no mutations were found, *GJC2* sequencing was used. Since 2013, targeted enrichment-based next-generation sequencing with 104 genes related with leukoencephalopathies or whole-exome sequencing were adopted for patients without definite genetic findings[46]. The novelty of the variations was examined using the dbSNP (https://www.ncbi.nlm.nih.gov/projects/SNP/), HGMD (http://www.hgmd.cf.ac.uk/), ExAC (http://exac.broadinstitute.org/), and 1000G (http://www.internationalgenome.org/) databases. To evaluate probably pathogenic variations, analysis of the amino acid sequence conversation, family segregation, and verification on 100 normal alleles were performed. In silico prediction including mutationtaster (http://www.mutationtaster.org), Polyphen-2 (http://genetics.bwh.harvard.edu/pph2/), SIFT (http://sift.jcvi.org/), or HSF (http://umd.be/HSF3/) were used. For patients without positive NGS results, chromosomal abnormality was screened using high-resolution G-banding chromosome analysis on PHA-stimulated circulating lymphocytes (SRL, inc).

## Results

Detailed clinical and genetic information of the 119 patients recruited is provided in S1 Table. Nine HMDs were identified in this sample, including PMD (94, 79%), Pelizaeus-Merzbacher-like Disease (PMLD, 10, 8%), hypomyelination with atrophy of the basal ganglia and cerebellum (HABC, 3, 3%), GM1 gangliosidosis (5, 4%), GM2 gangliosidosis (3, 3%), trichothiodystrophy (1, 1%), Pol III-related leukodystrophy (POL3R, 1, 1%), hypomyelinating leukodystrophy type 9 (HLD9; 1, 1%), and chromosome 18q deletion syndrome (18q- syndrome, 1, 1%) (Table 2). MRI scans were obtained from 69 patients, 48 of which were taken in patients over one year of age (Table 3, detailed information is summarized in S2 Table). Genetic aetiologies were identified in 112 (94%), revealing one chromosomal 18q terminal deletion and mutations in *PLP1* (89, 75%), *GJC2* (8, 7%), *TUBB4A* (3,3%), *GLB1* (5, 4%), *HEXA* (2, 2%), *HEXB* (1, 1%), *ERCC2* (1, 1%), *POLR3A* (1, 1%), and *RARS* (1, 1%) (Table 4). Thirteen *de novo* mutations were found, and eighteen variations from *PLP1*, *GJC2*, *TUBB4A*, *GLB1*, *HEXA*, *HEXB*, *ERCC2*, *POLR3A*, and *RARS* were novel. The information of pathologic validation for novel variations is provide in S3 Table and S4 Table. Seven patients (7, 6%) including P90-P94 with PMD and P103-P104 with PMLD were failed to diagnose genetically. In 112 patients diagnosed genetically, 66 with *PLP1* duplication or triplication mutations were discovered by MLPA, 31 with mutations in *PLP1*, *GJC2*, *HEXA*, *HEXB*, were discovered by Sanger sequencing, and 14 patients were found mutations in *PLP1*, *TUBB4A*, *GLB1*, *ERCC2*, *POLR3A*, and *RARS* by NGS.

**Table 2:** Clinical findings of 119 patients with hypomyelinating disorders.

		PMD	PMLD	HABC	GM1	GM2	TD	POL3R	HLD9	18q del
Clinical diagnosis		94	10	3	5	3	1	1	1	1
Genetic diagnosis		89	8	3	5	3	1	1	1	1
Gender										
	Male	93	4	1	1	1	-	-	-	-
	Female	1	6	2	4	2	1	1	1	1
Onset age										
	At birth	28	3	1	-	-	1	-	-	-
	0-1y	65	7	2	4	3	-	1	1	1
	>1y	1	-	-	1	0	-	-	-	-
Initial symptom										
	Nystagmus	85	7	1	-	-	-	-	1	-
	Development delay	8	3	2	4	2	-	1	-	1
	Pruritic ichthyosis	-	-	-	-	-	1	-	-	-
	Developmental regression	-	-	-	1	1	-	-	-	-
	Stridor	1	-	-	-	-	-	-	-	-
Development delay		94	10	3	5	3	1	1	1	1
Nystagmus		93	9	3	1	2	-	1	1	1
Muscle tone										
	Hypotonia	60	2	1	3	2	-	-	1	NA
	Normal	5	0	-	1	1	-	-	-	NA
	Hypertonia	19	5	2	1	1	1	1	-	NA
Increased DTR		27	5	1	1	1	NA	NA	NA	NA
Quadriplegia		24	3	1	1	2	1	0	0	0
Ataxia		23	3	0	1	0	0	1	NA	0
Tremor		17	0	2	1	1	0	1	0	0
Development regression		7	6	0	4	3	1	0	0	0
Convulsion		10	2	1	1	1	0	0	0	0
Visual abnormality		24	1	1	0	0	0	1	NA	0
Hearing abnormality		9	1	0	2	0	0	1	NA	0
Dysarthria		11	NA	1	0	2	0	NA	NA	NA
Swallowing difficulty		10	NA	0	0	1	0	0	0	0

PMD, Pelizaeus-Merzbacher disease; PMLD, Pelizaeus-Merzbacher like disease; HABC, hypomyelination with atrophy of the basal ganglia and cerebellum; GM1, GM1 gangliosidosis; GM2, GM2 gangliosidosis; TD, trichothiodystrophy; POL3R, Pol III-related leukodystrophy; HLD9, hypomyelinating leukodystrophy type 9; 18q del, chromosome 18q deletion syndrome; DTR, deep tendon reflex; NA, not acquired.

**Table 3:** Findings of brain MRI in patients>1y with hypomyelinating disorders.

MRI>1y		PMD	PMLD	HABC	GM1	GM2	POL3R	HLD9	18q del
No. Enrolled		35	4	3	4	1	1	1	1
T2 hyperintensity									
	Homogeneous	22	2	3	0	1	0	0	0
	Subcortical white matter	35	4	3	3	1	0	1	0
	Deep white matter	34	4	3	1	1	1	1	1
	Periventricular white matter	31	4	3	1	1	1	1	1
	Optic radiation	9	3	3	1	1	0	1	0
	Genu of corpus callosum	19	3	2	0	1	0	0	0
	Splenium of corpus callosum	17	3	2	0	1	0	0	0
	Pons (part)	9	4	0	0	0	1	0	0
	Pyramidal tracts of midbrain	3	4	1	0	0	0	1	0
	Anterior limb of internal capsule	26	2	0	0	1	1	1	0
	Posterior limb of internal capsule	23	4	2	2	1	1	1	0
	Substantia nigra	1	0	0	0	0	0	0	0
T2 hypointensity									
	Anterolateral part of thalamus	30	3	3	3	1	1	1	1
	Globus pallidus	3	2	3	1	0	0	0	1
Atrophy									
	Cerebellum	2	2	3	3	1	0	0	0
	Cerebrum	0	0	3	0	0	0	1	0

PMD, Pelizaeus-Merzbacher disease; PMLD, Pelizaeus-Merzbacher like disease; HABC, hypomyelination with atrophy of the basal ganglia and cerebellum; GM1, GM1 gangliosidosis; GM2, GM2 gangliosidosis; POL3R, Pol III-related leukodystrophy; HLD9, hypomyelinating leukodystrophy type 9; 18qdel, chromosome 18q deletion syndrome.

**Table 4:** Molecular findings in patients with hypomyelinating disorders.

Patient ID	Gene	Variation 1				Variation 2			
		Nucleotide Change	Amino acid Change	Parental derivation	Novel/ Reported	Nucleotide Change	Amino acid Change	Parental derivation	Novel/ Reported
P1-P20,P22-P64	*PLP1*	Duplication		Maternal					
P021,P065	*PLP1*	Duplication		*de novo*					
P066	*PLP1*	Triplication		Maternal					
P067	*PLP1*	c.517C>T	p.P173S	Maternal	Reported				
P068	*PLP1*	c.709T>G	p.F237V	*de novo*	Reported				
P069	*PLP1*	c.623G>T	p.G208V	Maternal	Reported				
P070	*PLP1*	c.353C>G	p.T118R	Maternal	Reported				
P071	*PLP1*	c.646C>T	p.P216S	*de novo*	Reported				
P072	*PLP1*	c.467C>T	p.T156I	*de novo*	Reported				
P073	*PLP1*	c.96C>G	p.F32L	Maternal	Reported				
P074	*PLP1*	c.457C>T	p.T156I	Maternal	Reported				
P075	*PLP1*	IVS5-1G>A		*de novo*	Reported				
P076	*PLP1*	c.623G>A	p.G208D	Maternal	Novel				
P077	*PLP1*	c.391C>T	p.Q131*	*de novo*	Reported				
P078	*PLP1*	c.97T>C	p.C33R	Maternal	Novel				
P079	*PLP1*	c.614G>A	p.R205K	Maternal	Novel				
P080	*PLP1*	c.111_119delTGAAGCCCT	p.E38_L40del	Maternal	Reported				
P081	*PLP1*	c92T>C	p.L31P	Maternal	Reported				
P082	*PLP1*	c.743C>A	p.A248E	*de novo*	Reported				
P083	*PLP1*	c.515T>C	p.V172A	*de novo*	Reported				
P084	*PLP1*	c.718T>C	p.F240L	Maternal	Novel				
P085	*PLP1*	c.535A>C	p.N179H	Maternal	Novel				
P086	*PLP1*	c.670_672delCTT	p.L224del	Maternal	Reported				
P087	*PLP1*	c.508T>C	p.S170P	Maternal	Reported				
P088	*PLP1*	c.552C>G	p.C184W	Maternal	Reported				
P089	*PLP1*	c.613A>G	p.R205G	Maternal	Reported				
P095	*GJC2*	c.925_938delCCCGCCCCCGCGCC	p.P309Afs*34	Paternal	Novel	c.201C>G	p.C67T	Maternal	Novel
P096	*GJC2*	c.689delG	p.G230Afs	Maternal	Reported	c.735C>A	p.C245*	Paternal	Reported
P097	*GJC2*	c.579delC	p.P194Rfs*16	Paternal	Reported	c.1296_1297insG	p.G433Gfs*59	Maternal	Reported
P098	*GJC2*	c.579delC	p.P194Rfs*16	Paternal	Reported	c.1296_1297insG	p.G433Gfs*59	Maternal	Reported
P099	*GJC2*	c.216delGinsAA	p.P73Tfs*35	Paternal	Reported	c.216delGinsAA	p.P73Tfs*35	Paternal	Reported
P100	*GJC2*	c.138C>G	p.I46M	Paternal	Reported	c.138C>G	p.I46M	Maternal	Reported
P101	*GJC2*	c.814T>G	p.Y272D	*de novo*	Reported	c.814T>G	p.Y272D	*de novo*	Reported
P102	*GJC2*	c.217C>T	p.P73S	Paternal	Reported	c.217C>T	p.P73S	Maternal	Reported
P105	*TUBB4A*	c.785G>A	p.R262H	*de novo*	Reported				
P106	*TUBB4A*	c.1190G>T	p.W397L	*de novo*	Novel				
P107	*TUBB4A*	c.538G>A	p.V180M	*de novo*	Novel				
P108	*GLB1*	c.622C>T	p.R208C	Maternal	Reported	c.520T>C	p.Y174H	Paternal	Reported
P109	*GLB1*	c.550C>T	p.Q184*	Paternal	Novel	c.446C>T	p.S149F	Maternal	Reported
P110	*GLB1*	c.1703A>G	D568G	NA	Novel	c.245C>T	T82I	NA	Reported
P111	*GLB1*	c.1343A>T	p.D448V	Paternal	Reported	c.1343A>T	p.D448V	Maternal	Reported
P112	*GLB1*	c.424A>T	p.K142*	Paternal	Reported	c.424A>T	p.K142*	Maternal	Reported
P113	*HEXA*	c.1031T>C	p.F344S	Maternal	Novel	IVS5-1G>T		Paternal	Reported
P114	*HEXA*	c.1031T>C	p.F344S	Maternal	Novel	IVS5-1G>T		Paternal	Reported
P115	*HEXB*	c.64_65delCT	p.L22Afs*92	Paternal	Novel	c.335_359delATCATGAACCTGCTGAATTCCAGGC	p.H112Lfs*34	Maternal	Novel
P116	*ERCC2*	c.2164C>T	p.R722W	Maternal	Reported	c.1808_1809delAA	p.K603Sfs*45	Paternal	Novel
P117	*POLR3A*	c.2722G>T	p.D908Y	Maternal	Novel	c.200G>A	p.R67H	Paternal	Novel
P118	*RARS*	c.5A>G	p.D2G	Paternal	Reported	c.1625+2T>G		Maternal	Novel

GenBank accession number: *PLP1*, NM_000533.4; *GJC2*, NM_020435.3; *TUBB4A*, NM_006087.3; *GLB1*, NM_000404.2; *HEXA*, NM_000520.4; *HEXB*, NM_000521; *ERCC2*, NM_000400.3; *POLR3A*, NM_0007055.3; *RARS*, NM_002887.

NA, not acquired.

**Fig 1:**
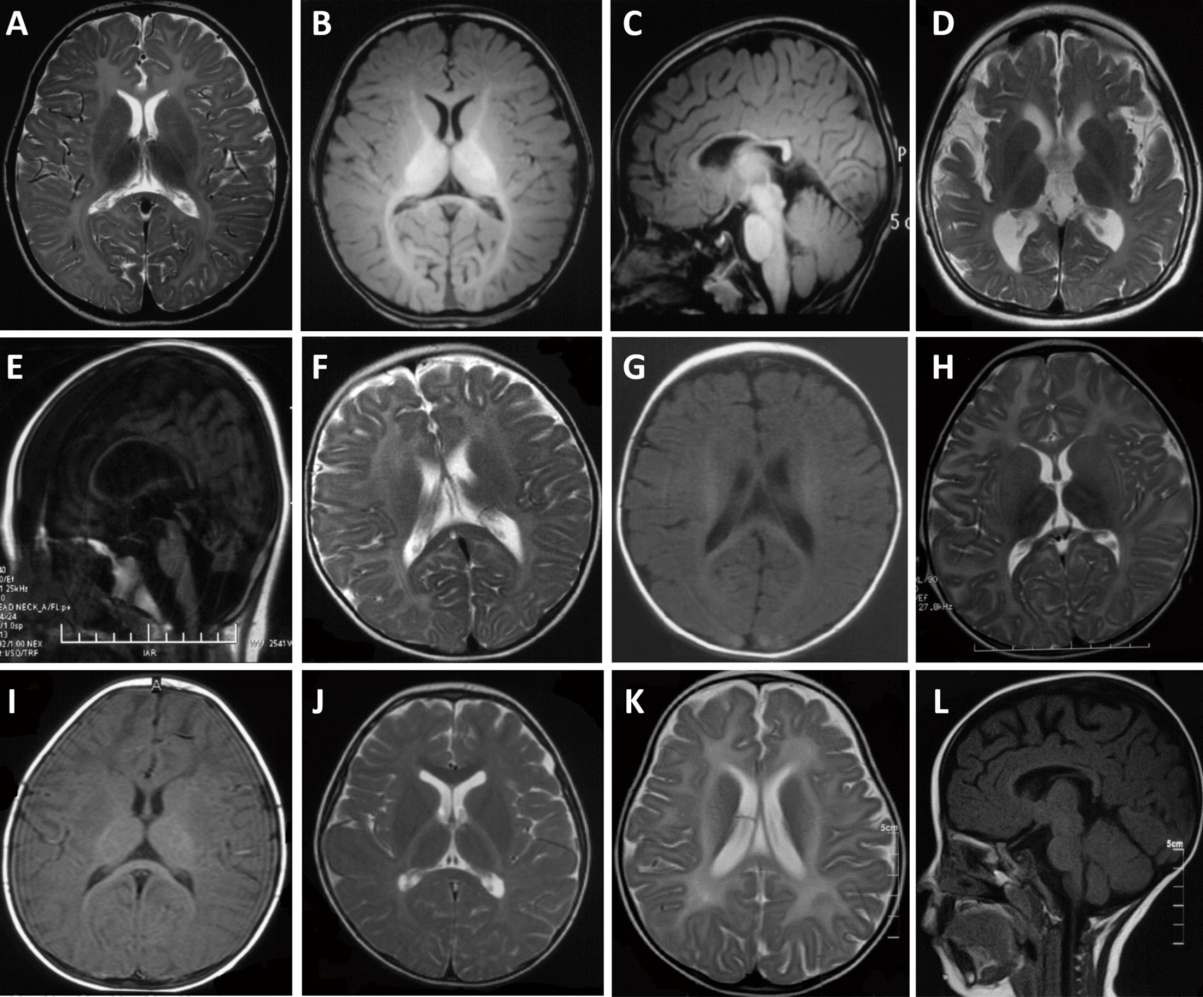
MRI findings of patients with hypomyelinating disorders. (A)–(C) P65 with PMD at 69 months of age. Homogeneous T2 hyperintensity in white matter and atrophy of corpus callosum is seen in axial T2WI (A) and sagittal T1WI (C). Axial T1WI (B) shows mild hyperintensity. (D)–(E) P106 with HABC at 7 years of age shows atrophy of cerebrum, relative preserved putamen, mild atrophy in caudate nucleus in axial T2WI (D), atrophy of cerebellum and corpus callosum in saggital T1WI (E). (F)–(G) P111 with GM1 gangliosidosis at 69 months of age showing T2 hyperintensity and cerebral atrophy. (H)–(I) P115 with GM2 gangliosidosis at 14 months old. T2WI shows diffuse hyperintensity in white matter and hypointensity in bilateral thalami (H), and mild hypointensity in white matter in T1WI (I). (J) Axial T2WI in P117 with Pol III-related leukodystrophy at 3 years of age showing diffuse T2 hyperintensity in deep white matter and posterior limb of internal capsules. T2 hypointensity was presented in anterior limb of internal capsules and corpus callosum. Axial T2WI (K) and saggital T1WI (L) of P118 with HLD9 at 18 months of age showing diffuse T2 hyperintensity in white matter and atrophy of cerebrum and corpus callosum.

Ninety-four patients (93 male and 1 female) were clinically diagnosed with PMD, of which 89 were found to have *PLP1* mutations. Patient 21 (P21) was diagnosed with PMD due to her classic phenotype and *de novo PLP1* duplication. She presented developmental delays starting at three months of age, and death occurred 20 months later. Onset of symptoms in 93 of the patients occurred during the first year of life, with 28 showing symptoms since birth. Initial symptoms of all 94 patients included nystagmus (85, 90%), developmental delay (8, 9%), and stridor (1, 1%). Developmental delay (94, 100%), nystagmus (93, 99%), and abnormal muscle tone (79, 94%) were the most commonly seen symptoms over the whole clinical course (Table 2). Among 35 MRI taken in patients after one year of age, homogeneous hypomyelination was found in 22 (63%). 3 (9%) were found T2 hyperintensity in pyramidal tracts of midbrain and T2 hypointensity in globus pallidus, respectively (Table 3; Fig 1A–1C).

89 (P1–P89) were found hemizygous or heterozygous *PLP1* mutations, including *PLP1* duplications in P1–P65, *PLP1* triplication in P66, point mutations, small deletions, and splicing site mutations in P67–P89. Splicing site mutation of c.697-1G>A (IVS5-1G>A) at the acceptor site of intron5 was found in P75. Hemizygous small deletions of c.111_119delTGAAGCCCT (p.E38_L40del) and c.670_672delCTT (p.L224del) were detected in P80 and P86, respectively. The same variation of c.467C>T (p.T156I) was found in P72 and P74. Different nucleotide alternations at c.623 were found in P69 (c.623G>T(p.G208V)) and P76 (c.623G>A(p.G208D)). Nine patients were identified with *de novo* mutations: P21 and P65 with genomic duplication, P68 with c.709T>G (p.F237V), P71 with c.646C>T (p.P216S), P72 with c.467C>T (p.T156I), P75 with c.697-1G>A (IVS5-1G>A), P77 with c.391C>T (p.Q131*), P82 with c.743C>A (p.A248E), and P83 with c.515T>C (p.V172A) (Table 4). Five novel variations—including c.623G>A (p.G208D) in P76, c.97T>C (p.C33R) in P78, c.614G>A (p.R205K) in P79, c.718T>C (p.F240L) in P84, and c.535A>C (p.N179H) in P85—were identified as pathologic (S3 Table, S4 Table). Analysis of XCI was performed for P21, and the result revealed a random pattern with a ratio of 56:44.

PMLD were diagnosed in four male and six female patients. Onsets occurred during the first year after birth (10, 100%). Developmental delay (100%), nystagmus (9, 90%), developmental regression (6,60%), and hypertonia (5,50%) were common (Table 2). Relative high percentage of abnormal MRI signal was found across the whole brain expect for substantia nigra (Table 3). Compound heterozygous novel mutations of c.925_938delCCCGCCCCCGCGCC (p.P309Afs*34) and c.201C>G (p.C67T) were found in P95 (Table 4). Four missense mutations (c.138C>G(p.I46M), c.814T>G(p.Y272D), c.217C>T (p.P73S), and c.201C>G (p.C67T)), five small deletions and/or insertions (c.925_938delCCCGCCCCCGCGCC (p.P309Afs*34), c.579delC (p.P194Rfs*16), c.1296_1297insG (p.G433Gfs*59), c.689delG (p.G230Afs), homozygous c.216delGinsAA (p.P73Tfs*35)), and one nonsense mutation (c.735C>A(p.C245*)) in *GJC2* were identified (Table 4). While corresponding heterozygous variation was only found in P99’s father but not her mothers, the homozygous mutation of c.216delGinsAA (p.P73Tfs*35) in *GJC2* in P99 had been proven to be caused by paternal UPD (uniparental disomy)[40]. A *de novo* homozygous mutation of c.814T>G (p.Y272D) in *GJC2* was found in P101[42].

HABC was identified in P105–P107. Onset of developmental delay or nystagmus occurred during the first year of life, followed by abnormal muscle tone, tremor, and other neurological signs, while ataxia was not reported in each patient (Table 2). Hypomyelination in white matter was found in each, while in the anterior limb of the internal capsule, all three patients showed relatively low T2 signal intensity. T2 hyper-hypo-hyperintensity stripes were present in the posterior limb of the internal capsule in P105. Atrophy of the corpus callosum, the cerebrum, and the cerebellum was found in each patient, while basal ganglia atrophy was only found in P106 with caudate nucleus atrophy. T2WI hypointensity was found in the corpus callosum in P105 (Table 3; Fig 1D and 1E). Heterozygous mutations including two novel (c.538 G>A (p.V180M), c.974G>T (p.W325L)) and one reported c.785G>A (p.R262H) in *TUBB4A* were identified (Table 4).

GM1 gangliosidosis was identified in five patients (P108–P112) including four females and one male (Table 2). In all five patients, four onset occurred during the first year of life. Developmental regression (4, 80%), hypotonia (3, 60%), and hearing abnormality (2, 40%) were common to observe (Table 2). β-galactosidase deficiency was found in all four patients (P108-109, 111–112 with lysosome enzymes tests (S6 Table). Hypomyelination was found in all four patients with brain MRI (Table 3, Fig 1F and 1G). All had *GLB1* mutations, including two novel mutations of c.550C>T (p.Q184*) and c.1613A>G (p.D538G) (Table 4, S3 Table).

GM2 gangliosidosis was diagnosed in P113–P115 with developmental abnormality and neurological deterioration. Cherry-red spots in the macula was seen in P113, P115 was observed hepatomegaly, while facial dysmorphism or skeletal dysplasia was not found in all (Table 2). Hexosaminidase A deficiency was seen in P113, hexosaminidase A and B deficiency was seen in P115 (S6 Table). Diffuse hypomyelination in white matter, and atrophy of the corpus callosum and cerebellum were found in P113 (Table 3; Fig 1H and 1I). P113 and P114 were a pair of siblings and both of them were found to have the compound heterozygous mutations of c.1031T>C (p.F344S) and c.570-1G>T (IVS5-1G>T) in *HEXA*, of which c.1031T>C (p.F344S) was novel. P115 was found to have novel mutations of c.64_65delCT (p.L22Afs*92) and c.335_359del (p.H112Lfs*34) in *HEXB* (Table 4, S3 Table).

Trichothiodystrophy was identified in P116. Widespread ichthyosis was present at birth, followed by developmental delay. Developmental regression appeared at eight years of age and quadriplegia presented two years after that. Brittle hair was also noted. No obvious photosensitivity and immunodeficiency was observed. Microcephaly with a head circumference of 48.5cm was revealed at 8 years of age (Table 2). Compound heterozygous mutations of c.2164C>T (p.R722W) and novel c.1808_1809del (p.K603Sfs*45) in *ERCC2* were detected (Table 4, S3 Table).

**Fig 2:**
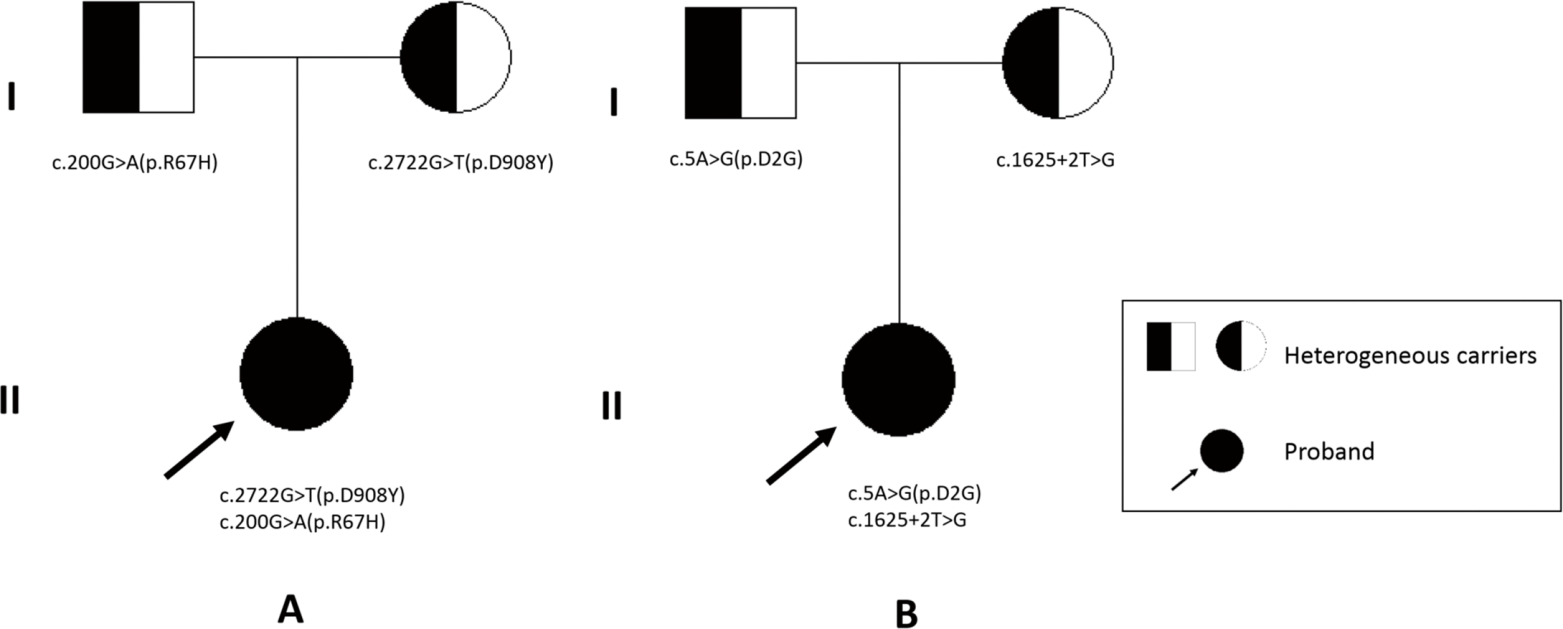
P117’s and P118’s pedigrees. (A) Compound heterozygous mutations of *POLR3A* c.2722G>T (p.D908Y) and c.200G>A (p.R67H) in P117. (B) Compound heterozygous mutations of *RARS* c.5A>G (p.D2G) and c.1625+2T>G in P118.

POL3R was diagnosed in P117. Mild motor development delay was presented after birth. She could walk without support at 2.5 years of age, but unsteadily and easily to fall. Then her motor function deteriorated and by 6 years of age, she was unable to walk or stand alone. After 2 years of age, intention tremor, nystagmus, vision deficiency, and startle reflex to sound was observed, and clinical examinations revealed intention tremor and spasticity of leg. She did not had abnormal dentition, and cognition was nearly normal (Table 2). Diffuse T2 hyperintensity in white matter except subcortical region was noted. It was also found in posterior limb of internal capsule and pons, while T2 signal in anterior limb of internal capsule, corpus callosum, globus pallidus, thalamus, and cerebellum was relatively normal (Fig 1J). Compound novel heterozygous mutations of c.2722G>T (p.D908Y) and c.200G>A (p.R67H) in *POLR3A* were identified (Table 4; Fig 2A).

HLD9 with *RARS* mutations was diagnosed in P118. Nystagmus was noted at three months of age. Motor development delay was severe, while cognition was relatively better preserved. She had hypotonia, involuntary head movement and spasticity, occasional tremor of legs, and myopia and incontinence were seen at five years of age (Table 2). Her head circumference at 19 months of age was 47.5cm. T2 hyperintensity was noted in optic radiation, pyramidal tracts of midbrain, and internal capsule with sparing of corpus callosum. Atrophy in cerebrum and corpus callosum was prominent (Table 3; Fig 1K and 1L). Compound heterozygous mutations were identified as c.5A>G (p.D2G) and novel c.1625+2T>G in *RARS*, of which the later was a substitution at the donor site of exon 13 (Table 4; Fig 2B).

**Fig 3:**
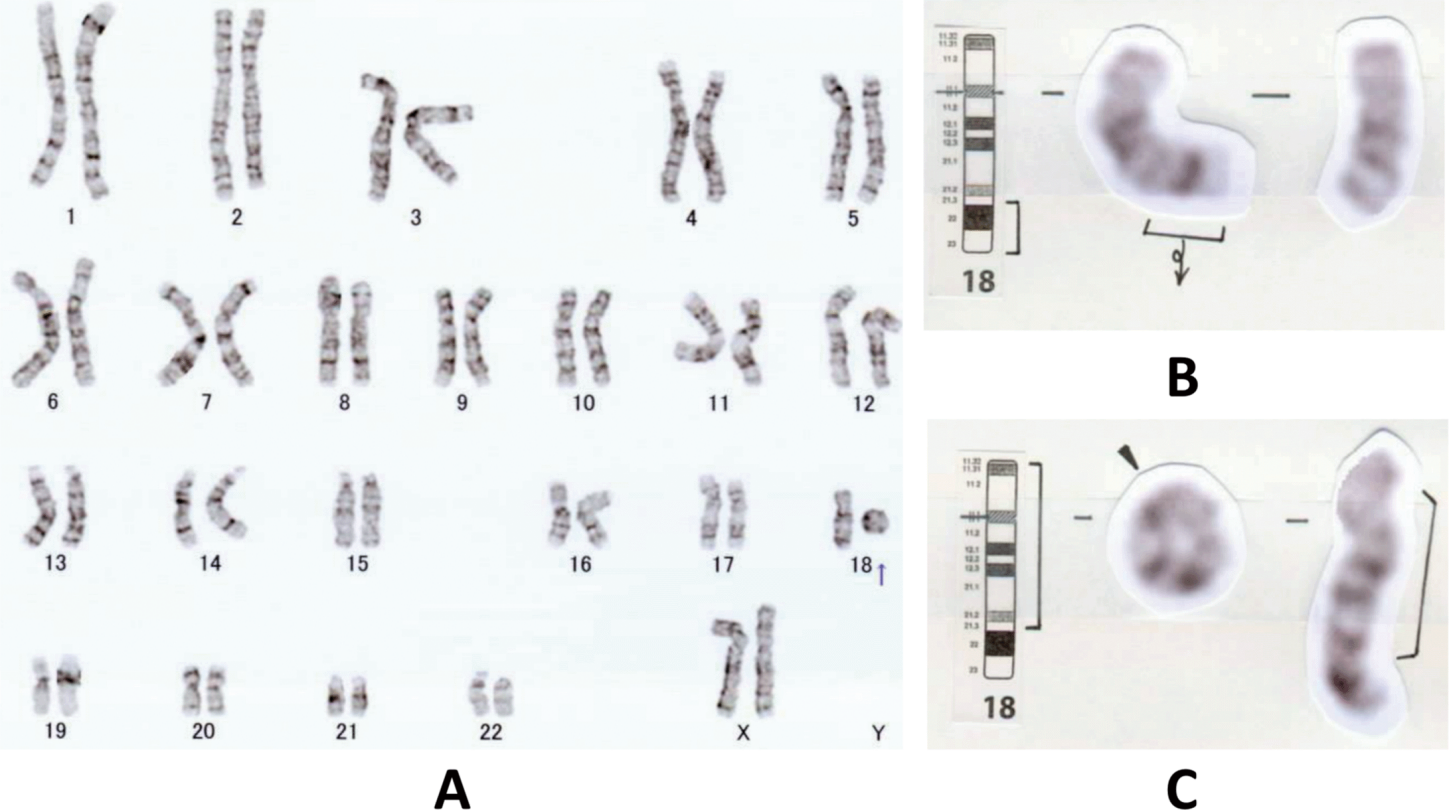
High-resolution G-banding chromosome analysis for P119 with chromosome 18q deletion syndrome. (A) A mosaic karyotype of 46,XX,del(18)(q21.3)/46,XX,r(18)(p11.32q21.3)/45,XX,-18 of P119. (B) Detailed illustrations of 46,XX,del(18)(q21.3). (C) Detailed illustrations of 46,XX,r(18)(p11.32q21.3).

18q- syndrome was diagnosed for P119, a girl showing developmental delay, nystagmus, and malformation including microcephaly, long philtrum, hypertelorism, and small hands and feet. MRI revealed mild T2 hyperintensity and supratentorial cerebral atrophy (Table 3). Chromosomal karyotype analysis on 20 cells revealed a mosaic condition of 46,XX,del(18)(q21.3)[9]/46,XX,r(18)(p11.32q21.3)[9]/45,XX,-18[2] in P119 (Fig 3). Her mother’s karyotype was 46,XX[27]/47,XXX[3] while her father’s was normal. No abnormal clinical symptoms were described for her parents.

## Discussion

HMDs are a growing group of heterogeneous, often progressive disorders with a wide range of symptoms and complications caused by a deficit in myelin deposit in the white matter. Clinical diagnosis of HMDs is based on symptoms and MRI expression. Developmental delay, nystagmus, abnormal neurological signs were seen in most patients. Congenital cataract in HLD5, hypodontia in Pol III-related leukodystrophies, ichthyosis and photosensitivity in trichothiodystrophy, or malformation in chromosome 18q deletion syndrome, deficiency of acid β-galactosidase, β-hexosaminidases A, or β-hexosaminidases B in GM1 or GM2 gangliosidosis are suggestive of diagnosis. Diffuse hypomyelination in white matter is characteristic of HLDs. Homogeneous T2 hyperintensity is seen in PMD, atrophy of putamen, caudate nuclei, and cerebellum is seen in HABC. Molecular diagnosis method is chosen based on clinical diagnosis. *PLP1* MLPA or sequencing are taken for patients with PMD, *GLB1* sequencing is taken for those with deficiency of acid β-galactosidase. In recent years, targeted enrichment-based next-generation sequencing or whole exome sequencing were used widely in diagnosing hypomyelinating leukodystrophy[3, 47], and were taken in this study for those with negative result of targeted gene sequencing. For patients with negative results of NGS, chromosomal analysis is suggested.

In this study, the HMDs found in the 119 enrolled patients included PMD, PMLD, HABC, GM1 and GM2 gangliosidosis, trichothiodystrophy, POL3R, HLD9, and 18q- syndrome (Table 2), of which POL3R and HLD9 were first diagnosed in China. One hundred and twelve patients were found to have genetic aetiologies, including 111 with mutations in *PLP1*, *GJC2*, *GLB1*, *TUBB4A*, *HEXA*, *HEXB*, *ERCC2*, *POLR3A*, and *RARS*, and 1 with deletion in chromosomal 18q terminal. In contrast to previous studies, mutations in *GLB1*, *HEXA*, *HEXB*, *ERCC2*, *POLR3A*, and *RARS* were found, revealing a positive molecular diagnostic rate of 94% (Table 5). A total of 18 novel mutations were discovered in this study, expanding the mutation spectrum (Table 3, S3 Table). Thirteen patients carried *de novo* mutations in *PLP1*, *GJC2*, or *TUBB4A*, of which *GJC2* c.814T>G (p.Y272D) in P101 was homozygous.

**Table 5:** Researches on the genetic heterogeneity of hypomyelinating disorders.

	This study	Numata et al. 2014	Arai-Ichinoi et al. 2015
Clinical diagnosis	119	101	26
Onset age	0-5y (97%<1y)	0-1y (91%<6m)	0–3y (median = 4m)
Initial symptoms			
Nystagmus	80%	56%	19%
Developmental delay	17%	34%	54%
Genetic diagnosis	112	49 (76 received molecular testing)	15
*PLP1*	75%	62%	12%
18q deletion	1%		12%
*TUBB4A*	3%		8%
*TREX1*			4%
*POLR3A*	1%		
*POLR3B*			4%
*KCNT1*			8%
*MCOLN1*			4%
15q loss of heterozygosity			4%
*GJC2*	7%		
*GLB1*	4%		
*HEXA*	2%		
*HEXB*	1%		
*ERCC2*	1%		
*RARS*	1%		
Molecular positive rate	94%	64%	58%

PMD caused by *PLP1* mutations is the most commonly recognized HMD. *PLP1* spans a ~17kb genomic interval, containing seven exons. It encodes PLP1 with 276 amino acids and its splicing isoform, DM20. Thirty-five amino acids, ranging from p.116 to p.150 of PLP1 (*PLP1*-specific region, or exon 3B), are spliced out in DM20. Both PLP1 and DM20 were important myelin-forming proteins in the white matter, however, the distribution and function of PLP1 and DM20 are different and DM20 alone cannot fully compensate for a lack of PLP1 in *PLP1* knockout mice[48–50]. Mutations in *PLP1* included genomic duplication, genomic deletion, point mutation, and mutations in splicing sites and noncoding regions—of which duplication and point mutations account for 60–70% and 15–20%, respectively[48, 51]. In our cohort, with 89 patients with *PLP1* mutations, a similar distribution of mutations of 66 genomic duplications, 20 point mutations, 2 small deletions, and 1 splicing site mutation was found. Genomic duplication was most common, accounting for 74%, while 22% were point mutations.

PLP1 is a tetra-span membrane protein with both N and C termini located in the cytoplasm. Three intracellular domains (ICD), four transmembrane domains (TMD), and two extracellular domains (ECD) in PLP1 protein are recognized, of which the *PLP1*-specific region is in ICD2. It was proposed that missense mutations in TMDs exhibit a severe phenotype, while phenotype of mutations in ECDs and in the *PLP1*-specific region were mild[52]. In 20 studies on the *PLP1* point mutations (S5 Table), mild PMD phenotype were found in 17/18 mutations in the *PLP1-*specific region, including frameshift and truncating mutations. Apart from splicing mutations, truncating mutations, and mutations in the *PLP1*-specific region and noncoding regions, severe phenotypes were observed in 49/82 amino acid substitution mutations, including 2/4 in ICD2, 7/9 in ECD1, 16/38 in ECD2, 9/13 in TMD1, 5/6 in TMD2, 6/8 in TMD3, and 4/4 in TMD4. In our research, among 20 point mutations and two small deletions, three were in TMD1, one was in the ECD1, two were in the ICD2, five were in the TMD3, ten were in the ECD2, and one was in the TMD4. Six out of eight mutations in TMDs and seven out of eleven mutations in ECDs were severe connatal PMD, and all three mutations in ICDs showed milder classical phenotype. p.T118R in P70 and p.Q131* in P77 in the *PLP1*-specific region were related to relatively mild classical phenotype. It could be concluded that, in general, severe phenotypes would have mutations in all four TMDs and ECD1, both mild and severe PMD phenotypes would occur from mutations in ICD2 and ECD2, and mild PMD phenotypes would present with mutations in the *PLP1*-specific region.

In general, only males are affected with PMD and females with *PLP1* mutations are normal or presented with mild PMD phenotype, following the X-linked recessive inheritance pattern. Occasionally, severe PMD phenotypes were found in females carrying *PLP1* mutations, probably attributed to abnormal XCI pattern, other dosage sensitive genes in the duplicated regions, or a changed expression pattern of *PLP1* or other genes. For carriers with *PLP1* duplications, random or moderately skewed XCI patterns were found in those with PMD phenotype and extremely skewed patterns were found in those with normal phenotype[53–56]. In our study, a female patient, P21, with *de novo PLP1* duplication, was found to be of classic PMD phenotype. Her phenotype and genetic findings in her and her family lead to the diagnosis of PMD, while a random XCI pattern revealed may suggest the reason why she was severely affected.

*TUBB4A* encodes a member of the beta tubulin family, which is important for the assembling of microtubules, and is expressed mainly in the brain, especially in the cerebellum, putamen, and white matter[57, 58]. Mutations in *TUBB4A* cause HABC with developmental delay, hypotonia, nystagmus, and deterioration of motor function. Hypomyelination, atrophy of the basal ganglia, and cerebellar and/or cerebral atrophy are characteristic in MRI scans[13]. More than 30 mutations in *TUBB4A* have been identified to date (http://www.hgvs.org/). In our study, atrophy of the putamen was absent in MRI scans in P105 (15 months), P106 (92 months), and P107 (62 months). It was reported that a normal putamen was presented in 31% patients younger than 2 years, and 97% patients between 2 and 12 years were found to have small or absent putamen[13]. In another study, a preserved putamen was found in five patients ranging from 4 to 45 years of age[59]. Therefore, atrophy of putamen may appear late or be absent in HABC, and a preserved putamen should not rule out the diagnosis of HABC. The T2 hyper-hypo-hyperintensity stripes in the posterior limb of the internal capsule found in P105 were also reported in another study in 2015, suggesting that these stripes may be a common sign in HABC[60]. Genetic diagnosis was made by findings of *TUBB4A* mutations, including c.538 G>A (p.V180M), c.974G>T (p.W325L), and c.785G>A (p.R262H), of which c.538 G>A (p.V180M) and c.974G>T (p.W325L) were novel. The mutation c.785G>A (p.R262H), found in P105, was also reported in another HABC patient in 2014, suggesting that it may be a disease causing mutation[61].

Lysosomal storage disorders (LSD) were presented with overlapping clinical features including developmental delay, ataxia, dysmorphic features, organomegaly, hydrocephaly, and skeletal dysplasia, resulting from the deficiency of lysosomal enzymes[62]. Hypomyelination was reported in several LSDs, including GM1 gangliosidosis with deficiency in acid β-galactosidase due to *GLB1* mutations, and GM2 gangliosidosis with deficiency in β-hexosaminidase A (Tay-Sachs disease, *HEXA* mutations) or β-hexosaminidases A and B (Sandhoff disease, *HEXB* mutations)[6, 38, 63–66]. In our study, development delay and hypomyelination were found in all eight patients (P108–P115) with GM1 gangliosidosis or GM2 gangliosidosis (Table 3, Fig 1F–1I). In addition, developmental regression, spasticity paraplegia, hyperacusis, Mongolian spots, hepatomegaly, and seizures were noted. Assessment of lysosome enzymes revealed β-galactosidase deficiency in four patients with GM1 gangliosidosis, hexosaminidase A deficiency in one patients with Tay-Sachs disease, and hexosaminidase A and B deficiency in one patient with Sandhoff disease. *GLB1* mutations in GM1 gangliosidosis, *HEXA* mutations in Tay-Sachs disease, and *HEXB* mutations in Sandhoff disease were found in all, of which *GLB1* c.622C>T (p.R208C) in P108 was a common mutation for patients with GM1 gangliosidosis[67]. *HEXA* IVS5-1G>T in P113 and P114 was reported by a previous study, in which the HEXA protein in the patient’s skin was undetectable[68]. Therefore, GM1 gangliosidosis or GM2 gangliosidosis could manifest as hypomyelination and a comprehensive analysis of lysosome enzymes, brain MRI, and genetic screening for precise diagnoses should be undertaken.

Trichothiodystrophy with *ERCC2* and *ERCC3* mutations is caused by defective DNA repair after UV damage, and hypomyelination was reported in trichothiodystrophy with *ERCC2* mutation[27, 69, 70]. In addition, hypomyelination was characterized in Cockayne syndrome, another disorder with *ERCC6* and *ERCC8* mutations also causing defective DNA repair after UV damage[69, 71]. In the present study, P116 with developmental abnormality, microcephaly, brittle hair, ichthyosis, spastic quadriplegia, and hypomyelination was found with c.2164C>T (p.R722W) and c.1808_1809del (p.K603Sfs*45) mutations in *ERCC2*. c.2164C>T (p.R722W) was reported in a boy with trichothiodystrophy in a 2011 study[26]. c.1808_1809del (p.K603Sfs*45) is a nonsense variation that may lead to truncation of the protein, and was predicted as pathologic (S3 Table).

POL3R was caused by a deficiency in RNA polymerase III complex due to biallelic mutations in *POLR3A*, *POLR3B*, or *POLR1C*[15, 72–74]. *POLR3A* mutations underlying POL3R was first reported in 2011[73, 74]. Hypomyelination, hypodontia and hypogonadotropic hypogonadism (4H syndrome) were characteristic, but clinical heterogeneity across populations was reported (S7 Table). Exacerbation after infections was reported in a Turkish and a Polish patient[75, 76]. A patient from southeastern Europe showed severe intellectual disability[77]. Seizures were rare but were reported in some Polish, French-Canadian, and European patients[73, 76, 77]. In a Japanese patient, no positive pyramidal signs, spasticity, cerebellar ataxia, tremor, nystagmus, oculomotor abnormalities, hypogonadotropic hypogonadism, or hypodontia were found[78]. In the present study, nystagmus occurred after an accident event, myopia, absence of hypogonadotropic hypogonadism or hypodontia, and startle reflex to sound showed in P117 were not common in most patients. The phenotype of P117 was similar to the Japanese patient, but obvious difference was noted when compared with patients in other western populations, which may reveal different clinical characteristics across different populations. In MRI scans, a combination of hypomyelination, T2 hypointensity of the thalami and/or the pallidi, T2 hypointensity of the optic radiations, and cerebellar atrophy showed a sensitivity of 85% and a specificity of 93% for identifying POL3R[79]. In our study, hypomyelination, T2 hypointensity of the thalami, globus pallidi, and optic radiations were seen in P117, while cerebellar atrophy was not observed (Fig 1J). On the other hand, similar to the T2 feature in the internal capsule in P117, it was reported in a 2016 study that T2 hyperintensity was present in the posterior limb with T2 hypointensity in the anterior limb[80], suggesting a untypical MRI sign in POL3R. Until now, more than 60 mutations were reported in *POLR3A*, including missense, nonsense, splicing site, small insertion, small deletion, and small insertion/deletion mutations (HGMD). In present study, two missense variations in *POLR3A*, c.2722G>T and c.200G>A, were identified. Both variations were predicted to be disease causing (S3 Table). Segregation analysis revealed that each of the parents carried one variant. Both variations affect evolutionarily conserved positions (S4 Table). c.2722G locates in the twentieth exon of *POLR3A*, and c.2722G>T resulted in an alternation of acidic aspartic acid to aromatic tyrosine (p.D908Y), in the fifth domain of RNA polymerases III, which is the discontinuous cleft required for forming the central cleft or channel to bind DNA[81, 82]. c.200G>A caused a translation of polar arginine to aromatic histidine in the first domain, which was supposed to play a role in positioning the DNA during transcription. Both variations could change the chemical property of RNA polymerases III and deteriorate the biochemical function of RNA polymerases III, thus were considered pathologic.

Hypomyelinating leukodystrophy caused by mutations of *RARS* was first reported in 2014[16],covering four Netherlandish patients including three mildly and one severely affected. They presented nystagmus, motor delay, intellectual disability, hypotonia, or spasticity. MRI revealed a hypomyelination pattern in white matter and atrophy of the corpus callosum, the cerebrum, and the cerebellum[16]. P118 in present study showed similar phenotype including neuroimaging findings (Fig 1K and 1L), while decreased visual acuity and incontinence were not reported.

Until now, five mutations in *RARS* were reported, including two missense mutations (c.5A>G (p.D2G), c.1535G>A (p.R512Q)), one splicing site mutation (c.45+1G>T), one nonsense mutation (c.96_97del (p. C32Tfs*39)) and one mutation changing the start codon (c.1A>G (p.Met1?)). In this study, compound heterozygous variations of c.5A>G (p.D2G) and novel c.1625+2T>G in *RARS* were identified. c.1625+2T>G was a substitution at the donor site of exon 13 and was predicted as affecting splicing and diseasing causing by in silico analysis (HSF, mutationtaster) (S3 Table). c.5A>G (p.D2G) in both studies was related to mild phenotype. Chromosomal 18q deletion was related with hypomyelination and considered one of the causes of the HLDs[1–5]. The clinical manifestations, location, and length of fragment lost varied greatly in different patients with chromosomal 18q deletion, and the hypomyelination may be caused by the haploinsufficiency of some genes, especially *MBP*, which is located in 18q23 and encodes myelin basic protein, one of the most important proteins constructing myelin[82–87]. P119’s karyotype was identified as 46,XX,del(18)(q21.3)/46,XX,r(18)(p11.32q21.3)/45,XX,-18, all with loss of the *MBP* region (Fig 3). However, it has been reported that a patient with a karyotype of 45,XX,-18/46,XX,r(18)(p11.3q23)/46,XX,dicr(18)(p11.3q23;p11.3q23), which indicated a loss of *MBP*, showed normal brain MRI findings[88], suggesting the hypomyelination was the result of a joint effort of many genes together in the fragment lost, not only MBP. In the presenting study, besides *MBP*, 46,XX,del(18)(q21.3) caused the loss of 16 morbid genes including *NEDD4L*, *MALT1*, *RAX*, *LMAN1*, *CCBE1*, *MC4R*, *PIGN*, *TNFRSF11A*, *BCL2*, *SERPINB7*, *SERPINB8*, *RTTN*, *CYB5A*, *TSHZ1*, *CTDP1*, *TXNL4A*, and 46,XX,r(18)(p11.32q21.3) were caused additional loss of *LPIN2* and *SMCHD1*. The phenotypes were related with immunodeficiency, growth abnormality, malformation, neurologic, cardiovascular, hematological, cutaneous, skeletal disorders, etc (Summarized in S8 Table). In contrast, only *TXNL4A*, the pathologic gene underlying Burn-McKeown syndrome with severe dysmorphism, was deleted in 46,XX,r(18)(p11.3q23) (https://decipher.sanger.ac.uk/excel, S8 Table). After all, the chromosomal aberrance may attribute to hypomyelination, and should be taken into consideration for a patient with HMD, especially with those with malformation or multisystem damage.

## Conclusion

In this study, nine different HMDs were diagnosed in 119 patients, of which 112 (94%) patients were found to have genetic aetiologies. This research was based on a large sample of patients with HMDs in a single center, and comprehensive clinical and genetic analysis were used. Targeted gene sequencing, targeted enrichment-based next-generation sequencing, whole exome sequencing, and chromosomal analysis were taken based on the phenotype, thus providing a better diagnostic strategies. HMDs with mutations in *POLR3A* and *RARS* were first identified in China.

## Supporting information

S1 Table Detailed clinical and genetic information of the 119 HMD patients recruited. (XLSX)

S2 Table Detailed MRI information of 48 HMD patients over one year of age. (XLSX)

S3 Table The information of pathologic validation for 18 novel variations in HMD patients. (XLSX)

S4 Table The information of conservation validation for 13 novel variations in HMD patients. (XLSX)

S5 Table The genotype and phenotype of *PLP1* point mutations from 20 studies. (XLSX)

S6 Table The results of lysosomal analysis for patients with GM1 and GM2 gangliosidosis. (XLSX)

S7 Table Comparison of the clinical manifestations between patients in the present study and other studies with *POLR3A* mutation. (XLSX)

S8 Table The morbid genes affected in P119 with chromosomal 18q deletion. (XLSX)
